# *Gastrodia elata*–Derived Parishin Extracts Attenuate Aging by Modulating Oxidative Stress, Inflammation, Apoptosis, and Metabolism

**DOI:** 10.1002/fsn3.71737

**Published:** 2026-04-03

**Authors:** Zhu Li, Yue Chen, Huihuang Shi, Peng Tang, Xiangui Zhang

**Affiliations:** ^1^ Yunnan Key Laboratory of Gastrodia and Fungi Symbiotic Biology, College of Agronomy and Life Sciences Zhaotong University Zhaotong China; ^2^ Yunnan Engineering Research Center of Green Planting and Processing of Gastrodia, College of Agronomy and Life Sciences Zhaotong University Zhaotong China

**Keywords:** apoptosis and metabolism, D‐galactose‐induced aging, inflammation, oxidative stress, parishins

## Abstract

This study investigates the anti‐aging properties of *Gastrodia elata*‐derived Parishin extracts (GEPE) by exploring its regulatory effects on oxidative stress, inflammatory responses, apoptosis, and metabolism. Network pharmacology identified 27 active components with parishins as core and 250 anti‐aging intersection targets of *Gastrodia elata*. Molecular docking confirmed strong binding activity of parishins to key targets such as EGFR, AKT1, and ALB. In vitro experiments showed that the GEPE concentration dependently reduced the activity of senescence marker SA‐β‐gal in D‐galactose‐induced senescent mouse microglial cell (BV2). In vivo experiments showed that GEPE improved cognitive and muscle functions, elevated organ indices of liver, thymus, and spleen, alleviated hepatic lipid accumulation and neuronal damage, reduced oxidative stress, inhibited inflammatory responses and cell apoptosis, and corrected metabolic disorders in D‐galactose‐induced aging mice. These findings demonstrate that GEPE is associated with attenuated aging, potentially through coordinated modulation of oxidative stress, inflammation, apoptosis, and metabolic homeostasis, highlighting its potential as a candidate for anti‐aging interventions.

## Graphical abstract

*Gastrodia elata*‐Derived Parishin Extracts (GEPE) attenuates aging by modulating oxidative stress, inflammation, apoptosis, and metabolisms, highlighting its potential as a candidate for anti‐aging interventions.

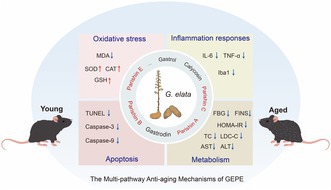

## 1 Introduction

Aging mainly refers to the degeneration of the structure and function of cells, tissues, and organs caused by advancing age (He and Sharpless 2017; López‐Otín et al. 2023). It is an irreversible natural process, involves complex interactions among multiple factors, including genetics, environment, and hormonal fluctuations, and is closely linked to the onset of various age‐related diseases (Rosoff et al. 2023; Widjaja et al. 2024; Argentieri et al. 2025). Currently, with the aging of the global population, age‐related diseases such as cardiovascular diseases, kidney disorders, and neurodegenerative diseases have increased significantly. This not only brings severe negative impacts on the quality of life of elderly people but also places an enormous economic burden on the whole society (Christensen et al. 2009; Li et al. 2021; Ye et al. 2023). The complexity of aging mechanisms and incomplete understanding of anti‐aging pathways have hindered the development of effective interventions, highlighting the need for investigating natural products with multi‐target regulatory potential.

It is widely recognized that targeting aging as a whole, rather than individual pathologies, may be a more effective strategy to address age‐related diseases and extend the healthspan of the elderly (Garmany et al. 2021). Cellular senescence is well‐known as a hallmark and major driver of aging, accumulating to accelerate tissue and organ deterioration, and is druggable for preventing age‐related comorbidities (Baker et al. 2016; He and Sharpless 2017; López‐Otín et al. 2023) (Xu et al. 2021). The oxidative stress and inflammation theories hold a prominent position in the onset and progression of aging as well as its associated diseases (Lin and Beal 2006; Kumar et al. 2023; Guzik and Touyz 2017). Many studies have shown that cellular oxidative stress responses are crucial factors accelerating cellular senescence (Tao et al. 2024; Wang et al. 2025) (Hauser et al. 2015; Snieckute et al. 2023). Cells under oxidative stress activate intracellular inflammatory signaling pathways, which then stimulate the secretion of inflammatory factors and bring about inflammatory responses (Matzkin et al. 2016; Chen, Ma, et al. 2025). Similarly, the excessive accumulation of inflammatory factors can disrupt normal intracellular metabolism, including the functionality of mitochondria, which in turn results in elevated generation of reactive oxygen species (ROS) and aggravated oxidative stress (Luk et al. 2023). Additionally, inflammatory factors can activate intracellular apoptotic signaling pathways (Luk et al. 2023). Oxidative stress generated during inflammation can further damage cellular DNA, mitochondria, and other organelles, initiating apoptotic mechanisms (Shen, Xu, et al. 2017). Metabolic disorders are widely recognized as key characteristics of aging (Xie et al. 2025). Excessive oxidative stress, persistent inflammation, and excessive apoptosis can all lead to metabolic disturbances, such as abnormal glucose and lipid metabolism, which accelerate the onset and progression of age‐related diseases and the aging process (Areloegbe et al. 2022). Therefore, a close, interactive, and dynamic relationship exists between metabolic disorders, inflammation, oxidative stress, and apoptosis. This dynamic interplay underscores the value of targeting these pathways collectively for anti‐aging interventions.

While aging represents an inevitable and irreversible biological process, the speed at which it progresses can be regulated. Traditional Chinese medicine (TCM)–derived molecules possess distinctive theoretical frameworks and extensive practical experience in the field of aging delay (Shen, Jiang, et al. 2017; Zumerle et al. 2024; Lee et al. 2025). Owing to their advantages of multiple components, multi‐target actions, multi‐signaling pathway regulation, diverse effects, and low toxicity, TCM aligns with the multi‐factor, multi‐mechanism, and multi‐theory characteristics of aging, making it a research hotspot in the field of anti‐aging drug development (Teng et al. 2025; Civiletto et al. 2025). *Gastrodia elata* Blume ( *G. elata* ), a perennial herb of the Orchidaceae family, is an important TCM with both medicinal and edible values, and its dried tubers are used clinically (Zhou, Han, et al. 2025). It has a wide range of clinical applications, including the treatment of epilepsy and convulsions, headache and dizziness, inability to move hands and feet, limb numbness, rheumatic arthralgia, infantile convulsions, and tetanus (Chen and Sheen 2011). In addition, preclinical studies have demonstrated that *G. elata* exhibits neuroprotective effects, improves learning and memory, exerts cardioprotective effects, regulates blood vessels, and has anxiolytic, anti‐fatigue, antioxidant, antidepressant, anti‐tumor, anti‐brain aging, and anti‐alcoholic liver injury activities (Gong et al. 2024, 2022; Huang et al. 2021; Zhou et al. 2024; Sun et al. 2025; Wang, Xin, et al. 2026). Based on its medicinal uses, numerous studies have investigated its active components, such as gastrodin, 4‐hydroxybenzyl alcohol, 4‐hydroxybenzaldehyde, bis‐(4‐hydroxybenzyl) sulfide, N6‐(4′‐hydroxybenzyl)‐adenosine, Parishin A, Parishin B, and Parishin C (Baek et al. 1999; Wang et al. 2024; Wang, Li, et al. 2026; Hsu et al. 2020, 2021; Jiang et al. 2024; Zhou, Chen, et al. 2025). Among these compounds, parishins are the main components of *G. elata* and exhibit effects such as relieving asthma, delaying aging, and antidepressant activity (Jang et al. 2010; Zhao et al. 2022; Jiang et al. 2024; Wang, Li, et al. 2026). However, the specific mechanism underlying their anti‐aging effect remains unclear.

This study aims to fill this gap by investigating the anti‐aging effects and underlying mechanisms of *Gastrodia elata*‐Derived Parishin Extracts (GEPE). We hypothesize that GEPE exerts anti‐aging effects by coordinately regulating oxidative stress, inflammatory responses, apoptosis, and metabolic homeostasis. To verify this hypothesis, we combined network pharmacology, molecular docking, in vitro (D‐gal‐induced BV2 microglial cell senescence model), and in vivo (D‐gal‐induced aging mouse model) experiments to systematically explore the active components, potential targets, and regulatory pathways of GEPE. The findings are expected to provide experimental evidence for GEPE as a potential aging inhibitor.

## 2 Materials and Methods

### 2.1 Screening of Active Components of *Gastrodia elata* and Their Potential Targets

Using “*Gastrodia elata*” as the keyword, TCMIP (http://www.tcmip.cn/TCMIP/index.php), HERB (http://herb.ac.cn/Search/), ETCM (http://www.tcmip.cn/ETCM/index.php/Home/), HIT (http://www.badd‐cao.net:2345), and SymMap (http://www.symmap.org/) databases were searched to collect chemical components of *G. elata* , with supplementary searches on CNKI and PubMed. The chemical components of *G. elata* were then retrieved from the PubChem database (https://pubchem.ncbi.nlm.nih.gov/), and files in “.sdf” format were downloaded, merged, and duplicates and components without structural information were removed. The CAS numbers or English names of the active components were searched in the TCMSP database (https://old.tcmsp‐e.com/tcmsp.php) to screen for active components of *G. elata* that met both the criteria of oral bioavailability ≥ 30% and drug‐likeness ≥ 0.18. For components not found in TCMSP, the SwissADME database (http://www.swissadme.ch/) was used for further screening, and components with high gastrointestinal (GI) absorption and two “YES” for drug‐likeness were selected. Subsequently, the “.sdf” format files of the active components were imported into the PharmMapper database (http://www.lilab‐ecust.cn/pharmmapper/) to obtain the top 300 predicted targets of the active components of *G. elata* (Wang et al. 2017). For components with no predicted targets, the SwissTargetPrediction database (http://www.swisstargetprediction.ch/) was used for further screening, with “ *Homo sapiens* ” as the species and Probability > 0 as the criterion. Components without target information were excluded. The target names collected from the two databases were converted to gene symbols, and redundant entries within this integrated dataset were filtered out. Through this sequential operation of data integration and duplicate elimination, the potential targets related to the active components of *G. elata* were finally acquired.

### 2.2 Screening of Potential Aging‐Related Targets

Using “Aging” as the keyword, potential aging‐related targets were retrieved from the GeneCards (https://www.genecards.org/), OMIM (https://omim.org/), and DrugBank (https://go.drugbank.com/) databases according to the previous literature (Zhou et al. 2020; Bisht et al. 2024). In the GeneCards database, a higher relevance score indicates a closer association between the target and the disease. Therefore, a screening process was conducted for disease‐related targets, with the application of a key criterion: relevance score > 1 and three rounds of screening using the median. The targets retrieved from the three aforementioned databases (GeneCards, OMIM, and DrugBank) were first combined into a single dataset. Subsequently, duplicate entries within this merged dataset were eliminated through systematic screening. This two‐step process—merging followed by deduplication—ultimately yielded a set of targets associated with potential aging‐related mechanisms.

### 2.3 Acquisition of Intersection Targets for Anti‐Aging Effect of *Gastrodia elata*

The targets corresponding to the active components of *G. elata* and the previously identified potential aging‐related targets were first uploaded to the Online Bioinformatics Analysis Platform. On this platform, a Venn diagram was generated through the built‐in analysis function, and this diagram was used to visually display the intersection targets that are associated with the anti‐aging effect of *G. elata* .

### 2.4 Construction of Protein–Protein Interaction (PPI) Network to Screen Key Anti‐Aging Targets of *Gastrodia elata*

The intersection targets of *G. elata* for anti‐aging were first uploaded to the STRING database (https://cn.string‐db.org/). During this database operation, the target species was specified as “ *Homo sapiens* ” and medium confidence (0.400) as the threshold. Isolated nodes were removed, and other parameters were set to default. The PPI network that had been constructed was subsequently imported into Cytoscape 3.9.0 software. This software was utilized to carry out the identification of key targets involved in the anti‐aging effect of *G. elata* .

### 2.5 GO Functional Annotation and KEGG Pathway Enrichment Analysis of Key Anti‐Aging Targets of *Gastrodia elata*

The DAVID database (https://david.ncifcrf.gov/summary.jsp) perform Gene Ontology (GO) and Kyoto Encyclopedia of Genes and Genomes (KEGG) enrichment analyses. These analyses targeted the intersection targets of *G. elata* for anti‐aging (Li, Yu, et al. 2025). The top 10 GO functions and top 10 KEGG pathways, ranked by *P*‐value (from smallest to largest), were visualized using the Online Bioinformatics Analysis Platform (http://www.bioinformatics.com.cn/plot_basic_gopathway_enrichment_bubbleplot_081).

### 2.6 Construction of *Gastrodia elata* Active Component‐Aging Target Network to Screen Key Anti‐Aging Active Components of *Gastrodia elata*

The intersection targets and active components of *G. elata* were organized in Excel according to data files and attribute files. The organized files were imported into Cytoscape 3.9.0 software to construct the *G. elata* active component‐aging target network. To identify the primary active components contributing to anti‐aging effects, the top 7 components with the highest degree values were selected as key anti‐aging active components.

### 2.7 Molecular Docking Verification of Key Anti‐Aging Active Components and Core Targets of *Gastrodia elata*

Molecular docking is a method for drug design that simulates the interaction modes between drugs and receptors like proteins and nucleic acids (Pinzi and Rastelli 2019; Li, Yu, et al. 2025). First, ligand file preparation was performed: the 3D structures of the key anti‐aging active components of *G. elata* in “.sdf” format were retrieved from the PubChem database and converted to “.pdbqt” format using Open Babel 2.4.1 software. Second, receptor file preparation was conducted: the crystal structures of the key anti‐aging targets of *G. elata* in “.pdb” format were downloaded from the RSCB PDB database (https://www.rcsb.org/), and crystal structures with high resolution (Å < 2.50) were selected for docking. Next, Autodock Vina 1.2.0 software was used for molecular docking, and calculations were performed using a genetic algorithm with all docking parameters set to default. Finally, PyMol 2.3.4 software was used to visualize some molecular docking results.

### 2.8 Preparation of *Gastrodia elata*‐Derived Parishin Extracts (GEPE)

2 kg of *G. elata* powder was subjected to reflux extraction with 20 L of 70% (w/w) ethanol three times at 75°C–80°C. The extraction durations were set to 3, 3, and 2 h in sequence. After completing the three rounds of extraction, the resulting extracts were merged into a single mixture. This mixture was then concentrated, and petroleum ether was added to the concentrated liquid for defatting treatment; this step was repeated until the extract achieved a colorless and transparent state. Subsequently, the separated aqueous phase was loaded onto a macroporous adsorption resin column for fractionation. Elution was performed using an ethanol‐water gradient system, with the following eluents applied in order: ultrapure water, 10% ethanol solution, 20% ethanol solution, 30% ethanol solution, 40% ethanol solution, 50% ethanol solution, 60% ethanol solution (used twice consecutively), 80% ethanol solution, 90% ethanol solution, and anhydrous ethanol. Subsequently, the 20% ethanol fraction was collected and further separated by silica gel column chromatography with a chloroform–methanol gradient elution (chloroform:methanol = 50:1 to 1:1, v/v). The crude parishin fraction was obtained from the eluate with a chloroform:methanol ratio of 3:1 (v/v). The crude parishin fraction was dissolved in chromatographic methanol and purified by preparative high‐performance liquid chromatography (preparative HPLC) with a mobile phase of acetonitrile‐water (15:85, v/v). A C18 reversed‐phase silica column was employed for the separation process, with the operating parameters set as follows: a flow rate of 15 mL/min and a column temperature maintained at 25°C. The eluate collected during this separation was then subjected to freeze‐drying treatment. This process yielded a white powdery crystal, which was formally defined as GEPE.

### 2.9 Cell Culture and Treatments

BV2 microglial cells were cultured according to a previously described protocol (Song et al. 2014). Briefly, the density of BV2 cells was observed under a microscope, and subculture was performed every other day when the cells reached confluence. Cells in 80% confluence with good growth status were used for experiments. First, BV2 cells were treated with D‐gal at concentrations of 0, 50, 100, 200, and 400 mM for 24 h to establish a D‐gal‐induced cellular senescence model. Subsequently, BV2 cells were cotreated with different concentrations of GEPE and 200 mM D‐gal for another 24 h.

### 2.10 Cell Viability Assay Using CCK‐8

Cell viability was assessed via a Super‐Enhanced Cell Counting Kit‐8 (CCK‐8) (Beyotime, C0048) according to a protocol adapted slightly from our prior publication (Chen, Yang, et al. 2025). In brief, digest logarithmic‐phase cells with trypsin, resuspend in 10% FBS medium to make single‐cell suspension, adjust concentration to an appropriate range (generally 5 × 10^4^ cells/mL). Next, 100 μL of cell suspension was dispensed into each well of a 96‐well plate. The plate was then placed in an incubator set at 37°C with 5% CO₂, and incubation was carried out for 20 h to facilitate cell attachment. Different concentrations of the corresponding drugs were then added, with 3 replicate wells per group, and incubation was continued for the corresponding time. Add 10 μL super‐enhanced CCK‐8 solution to each well. After that, the plate was placed in a cell incubator for 1 h of incubation. The 96‐well plate was then tapped lightly to ensure the orange formazan was mixed completely. Following this, a microplate reader was used to determine the absorbance (OD value) of each well at a wavelength of 450 nm.

### 2.11 β‐Galactosidase (SA‐β‐Gal) Staining

Cells were fixed and stained using a β‐galactosidase (SA‐β‐gal) staining kit (Cell Signaling, 9860) according to a previously described protocol with minor modifications (Kang et al. 2024). Briefly, cells were plated on coverslips in 12‐well plates, cultured normally for 24 h, then treated with respective drugs. Post‐treatment, medium was gently aspirated; each well was washed once with 1 mL 1 × PBS. Subsequently, 400 μL of SA‐β‐gal staining fixative was dispensed into each well, and fixation was performed at room temperature for 15 min. Once fixation was complete, the fixative was discarded, and each well was rinsed three times with 1 mL of 1 × PBS, with each rinse lasting 3 min. After aspirating the PBS solution, 400 μL of staining working solution was added to the wells. The cells were then incubated at 37°C overnight and later examined under a light microscope. Next, the activity of cellular SA‐β‐gal was further evaluated following the protocol provided by the manufacturer of the 96‐well Plate Cellular Senescence Assay Kit (Cell Biolabs, CBA‐231). A fluorescence microplate reader was used to detect fluorescence intensity, with the excitation wavelength set at 360 nm and the emission wavelength at 465 nm.

### 2.12 Animals

Male C57BL/6J mice (8 weeks of age, body weight ranging from 23 to 27 g) were acquired from Jiangsu Jicui Yaokang Biotechnology Co. Ltd. These mice were all housed in a specific pathogen‐free (SPF) animal breeding environment, where the housing conditions were controlled as follows: a 12‐h light–dark cycle (with the light phase lasting from 7:00 a.m. to 7:00 p.m.), a relative humidity of 55%, and an ambient temperature maintained at 21°C–23°C. During the housing period, the mice had unrestricted access to standard feed and drinking water. All experimental procedures were examined and authorized by the Animal Ethics Committee of Zhaotong University (approval number: 20220003A3), ensuring strict compliance with rigorous ethical standards. The sample size was set to 20 mice per group, which was determined based on preliminary experiments.

### 2.13 Animal Grouping and Drug Treatment

Following a 1‐week adaptive feeding period, the experimental mice were randomly assigned to two groups (control group and D‐gal‐induced group) using a random number table. For the D‐gal‐induced group, mice were administered subcutaneous injections of D‐gal at a dose of 200 mg/kg into the nape region, and this injection regimen was maintained for 8 consecutive weeks to construct an animal model of aging. In contrast, mice in the control group received subcutaneous injections of normal saline with an equivalent volume (0.1 mL/kg) over the same time frame. Once the aging model was successfully established, the mice in the D‐gal‐induced group were further subdivided into two subgroups: the aging model subgroup (Aging group) and the GEPE‐intervened subgroup (Aging + GEPE group). Mice in the Aging + GEPE group were given daily intragastric administration of GEPE at a concentration of 100 mg/kg. Meanwhile, mice in the Aging group were administered intragastric injections of normal saline with an equal volume (0.1 mL/kg), and both intervention protocols lasted for 8 consecutive weeks. Mice in the control group were gavaged with normal saline (0.1 mL/kg) for 8 consecutive weeks. Thus, the study included three groups: Normal control group, Aging group, and Aging+GEPE group. During the administration period, the mental state, diet, and skin and hair conditions of the mice were observed. After the administration, the mice were subjected to approximately 1 week of adaptive feeding before the formal experiment.

### 2.14 Behavioral Tests

Behavioral experiments were performed 1 week after the end of drug administration.

#### 2.14.1 Open Field Test

As referenced in previous study, the open field test was employed to assess the locomotor activity of the experimental mice (Lim et al. 2024). In brief, the experimental mice were randomly placed into a movement monitoring box (25 × 25 × 25 cm), and the total distance traveled by the mice within 10 min was recorded using a photoelectric sensor to assess their spontaneous exploratory behavior. Between successive tests, the movement monitoring box was first cleaned and then wiped with alcohol. This two‐step cleaning process was intended to eliminate any odor interference that might affect the experimental results.

#### 2.14.2 Y Maze Test

As referenced in a prior study with slight adjustments, the Y maze test was employed to assess the working memory (also referred to as short‐term memory) of the experimental mice (Yoshizaki et al. 2020; Hsu et al. 2021). In brief, the Y maze utilized in this experiment was composed of three identical arms, with each adjacent pair of arms forming a 120° angle. Each arm had specific dimensions: a length of 35 cm, a width of 5 cm, and a height of 15 cm. Experimental mice were positioned at the central area of the Y maze and permitted to explore the apparatus freely for a duration of 8 min. During the exploration period, two key parameters were recorded in real time via specialized software: the total number of times the mice entered any of the arms and the number of instances where the mice entered three distinct arms consecutively. To assess the working memory of the mice, the alternation percentage was computed using the following formula: Alternation percentage = [Number of non‐repetitive entries into the three arms/(Total number of arm entries − 1)] × 100%.

#### 2.14.3 Morris Water Maze Test

As described in our previous study with minor modifications, the Morris water maze test was used to evaluate the spatial cognitive function of the experimental mice (Li et al. 2020; Chen, Yang, et al. 2025). Briefly, taking advantage of the natural water aversion behavior in mice, the Morris water maze test was divided into three distinct phases: adaptive training, place navigation training, and spatial probe test. The Morris water maze was a circular pool with a diameter of 1 m and a height of 35 cm, with a visible platform fixed in a specific area. The swimming time, distance, and trajectory of the mice were recorded by video. Patterns of different colors and shapes (e.g., circles, squares) were attached to the walls of the pool in different quadrants as cognitive references. For adaptive training, non‐toxic and colorless warm water (23°C–25°C) was added to the pool. The visible platform was positioned such that it stood 1.5 cm above the water surface. Each mouse was placed into the pool with its head oriented toward the pool wall, and the duration taken for the mouse to reach the platform was documented. For mice that did not reach the platform within 90 s, a guide rod was used to direct them to the platform, and they were then allowed to remain on the platform for 30 s. Each mouse underwent training once in each of the four quadrants of the pool. Place navigation training commenced 1 day after the adaptive training phase and was carried out for five consecutive days. During this training, the platform was lowered to a depth of 0.5–1 cm below the water surface and rendered invisible by adding white dye to the water. Mice were placed into the pool from each quadrant, with their heads facing the inner wall, and a video recording system was used to capture their swimming trajectories and the latency (time taken) to reach the hidden platform. Mice that failed to locate the platform within 90 s were assigned a latency value of 90 s; afterward, they were guided to the platform and allowed to stay there for 30 s, which facilitated the evaluation of their cognitive memory. On the 7th day of the overall test protocol, the spatial probe test was conducted, and the platform was removed from the pool for this phase. Mice were placed into the pool from various quadrants and permitted to swim freely for 90 s. To assess the mice's spatial memory, three key parameters were recorded: their swimming trajectories, the number of times they crossed the original position of the platform, and the total time spent in the target quadrant (the area where the platform had been placed during previous training).

#### 2.14.4 Nest Building Test

The nest building test was conducted with slight modifications based on methods reported in prior studies (Deacon 2006; Hsu et al. 2021). Briefly, 1 h before the dark cycle, nesting material was placed into the mouse cage, and sufficient food and water were provided. After overnight incubation, the nesting results were observed and scored using the Nesting Score System (NSS). Two independent observers, who were kept unaware of the group allocations, were responsible for scoring the structure of the nests. The scoring system was divided into 5 major categories:(1) Score 0: Mice did not disturb or use the nesting material; (2) Score 1: Mice disturbed the material but did not aggregate it into a nest (material was simply piled); (3) Score 2: Mice aggregated the material into a flat nest with a shallow dish‐like appearance and incomplete or absent nest walls; (4) Score 3: Mice aggregated the material into a cup‐shaped nest with recognizable cup‐like or bowl‐like nest walls; (5) Score 4: Mice aggregated the material into an incomplete dome‐shaped nest with nest walls merging to form more than a hemisphere; (6) Score 5: Mice aggregated the material into a complete dome‐shaped nest with only one entrance/exit large enough for one mouse to enter and exit. During the nest building process, researchers were prohibited from entering the animal room to maintain a quiet environment and avoid disturbing the mice.

#### 2.14.5 Grip Strength Test and Endurance Test

Muscle strength and function gradually decline during aging, which is a natural physiological phenomenon. The forelimb grip strength and wire mesh climbing endurance tests were used to evaluate the muscle strength of mice in each group. For the grip strength test, mice were placed on the horizontal bar at the front of a grip strength meter, allowing their bodies to lie flat naturally with all four limbs grasping the bar. Once the mice had a firm grip, their tails were pulled horizontally backward, and the grip strength value displayed on the meter was recorded. Each mouse was tested 5 consecutive times; the maximum and minimum values were excluded, and the remaining maximum value was used for statistical analysis, followed by correction for body weight. If a mouse failed to grip firmly with all four limbs, turned its head, or struggled left/right, it was returned to the cage and allowed to calm down before re‐testing. For the endurance test, a self‐made circular wire mesh (diameter: 600 mm) was placed vertically, 60 mm above the water surface. Mice were placed on the wire mesh, and their endurance was evaluated based on the time until they fell into the water due to exhaustion from fear of drowning. For each group, mice with the highest and lowest body weights were excluded. Each mouse was tested 3 times with a 3‐min rest interval between tests. The longest time was recorded as the climbing time, followed by correction for body weight to represent the endurance of the mice.

### 2.15 Sample Preparation

After the behavioral tests, the experimental mice were fasted for 1 day in advance. The next day, mice were deeply anesthetized with carbon dioxide. Different samples were collected from each group. First, blood was collected from the orbital venous plexus of mice using capillary tubes, transferred to EP tubes, and allowed to coagulate at room temperature for 1 h. The samples were then centrifuged at 4000 rpm for 15 min at 4°C to separate the serum, which was stored at −80°C for subsequent analytical procedures. Meanwhile, the brain, spleen, thymus, and liver tissues of the experimental mice were rapidly harvested, and surrounding tissues were removed. The tissues were weighed immediately, rapidly frozen using liquid nitrogen and then stored at −80°C to be used for subsequent biochemical analytical tests. After orbital blood collection, the experimental mice were perfused with 20 mL of pre‐cooled 1 × PBS (pH 7.4) via the heart until the liver turned completely pale yellow. The collected tissues were subsequently fixed in a 4% paraformaldehyde (PFA) solution for a 48 h period, and this fixed sample was used for subsequent histological analytical procedures.

### 2.16 Determination of Organ Index

The liver is the main metabolic organ of the body, responsible for important physiological functions such as oxidation, glycogen storage, and protein synthesis (El‐Haskoury et al. 2018). The thymus and spleen are important immune organs of the body. After the behavioral experiments, mice were weighed, and the liver, spleen, and thymus were collected separately. The tissues surrounding the target organs were excised, after which the isolated organs were weighed accurately. To determine the organ index, the following formula was applied: Organ index (%) = (Weight of the organ/Body weight of the mouse prior to dissection) × 100%.

### 2.17 Hematoxylin–Eosin (H&E) Staining, Immunohistochemical (IHC) Staining, and TUNEL Staining

Hematoxylin–eosin (H&E) staining and immunohistochemical (IHC) staining were carried out in line with our previously reported experimental protocol, with slight adjustments made as needed (Li et al. 2024). In brief, tissue samples of the brain and liver were first fixed using 4% PFA. After the fixation process, the samples were embedded in paraffin wax and then sliced into sections with a thickness of 5 μm. For H&E staining, the paraffin sections underwent dewaxing and dehydration treatments sequentially, followed by staining in accordance with standard laboratory procedures. For IHC staining, this technique was specifically applied to assess the expression level of Iba1 (1:500, Wako, 019‐19,741) in brain tissue. The terminal deoxynucleotidyl transferase‐mediated dUTP nick‐end labeling (TUNEL) assay was performed using a TUNEL kit (Solarbio, T2196) to assess apoptosis in brain tissue cells. Finally, images were captured using a light microscope or fluorescence microscope and analyzed using ImageJ software.

### 2.18 Nissl Staining

Nissl staining was performed as previously described by us (Li et al. 2020). Briefly, paraffin‐embedded brain tissue samples were cut into 5‐μm‐thick sections. After being baked at 60°C for 40 min, the tissue sections were sequentially dewaxed in xylene I and xylene II, with each dewaxing step lasting 10 min. Following this, the sections were treated with absolute ethanol, 95% ethanol, and 80% ethanol in turn, with each ethanol treatment phase lasting 5 min. After the ethanol treatments, the sections were rinsed with PBS for a 10 min period. Next, the sections were placed in a toluidine blue solution and incubated at 60°C for 15 min, after which they were rinsed with PBS for 5 min. Routine dehydration was then conducted using a series of ethanol solutions: 80% ethanol for 5 s, 95% ethanol for 30 s, and absolute ethanol I and absolute ethanol II (1 min each). Once the dehydration process was complete, the sections were air‐dried and mounted on slides. Finally, a light microscope was used to observe neuronal damage in specific brain regions, including the cerebral cortex, hippocampal CA1 region, hippocampal CA3 region, and dentate gyrus (DG). The number of neurons in these aforementioned regions was counted, and the obtained data was determined and analyzed using ImageJ software.

### 2.19 Detection of Antioxidant Indexes and Inflammatory Cytokines in Serum, Brain, and Liver Tissues

The activities of four key oxidative stress‐related indicators—superoxide dismutase (SOD), glutathione (GSH), catalase (CAT), and malondialdehyde (MDA)—in mouse serum, brain tissue, and liver tissue were determined using respective commercial detection kits. The specific kits used were as follows: SOD kit (A001‐3‐2), GSH kit (A006‐1‐1), CAT kit (A007‐1‐1), and MDA kit (A003‐1‐2). All detection procedures were performed in strict accordance with the instructions provided by the kit manufacturer (Nanjing Jianchang Bioengineering Institute). Additionally, the levels of tumor necrosis factor‐α (TNF‐α, Solarbio, SEKM‐0034) and interleukin‐6 (IL‐6, Solarbio, SEKM‐0007) in serum, brain, and liver tissues were measured using commercial enzyme‐linked immunosorbent assay (ELISA) kits in accordance with the manufacturer's recommended guidelines and our previously described protocol (Li et al. 2024; Chen, Yang, et al. 2025).

### 2.20 Determination of Caspase‐3 and Caspase‐9 Activities in Brain Tissue

The activities of caspase‐3 and caspase‐9, two key enzymes involved in the apoptotic pathway, in mouse brain tissue were measured using specialized assay kits. Specifically, a Caspase‐3 Activity Assay Kit (Beyotime, C1116) and a Caspase‐9 Activity Assay Kit (Beyotime, C1158) were respectively utilized according to the manufacturer's instructions.

### 2.21 Detection of Blood Biochemical Metabolic Indicators

Serum samples from mice were used to detect the levels of fasting blood glucose (FBG), aspartate transaminase (AST), alanine transaminase (ALT), triglycerides (TG), total cholesterol (TC), and low‐density lipoprotein cholesterol (LDL‐C) using an automatic biochemical analyzer. The fasting insulin (FINS) level in the serum of experimental mice was determined via ELISA (Coibo Bio, CB12927‐Mu) method, with the assay procedure strictly following the instructions provided by the corresponding kit. To evaluate insulin resistance in the mice, the homeostasis model assessment of insulin resistance (HOMA‐IR) index was computed using the established formula: HOMA‐IR = [FBG (mmol/L) × FINS (mIU/L)] / 22.5.

### 2.22 Statistical Analysis

Outcome assessments, including histological staining (H&E, IHC, TUNEL, Nissl staining) and behavioral scoring (nest building test), were performed by two independent observers who were blinded to the group allocations to avoid subjective bias. For all datasets involving comparisons among the three experimental groups statistical analysis was conducted as follows: first, one‐way analysis of variance (ANOVA) was used to evaluate overall differences between groups; if a significant main effect was detected (*p* < 0.05), pairwise comparisons were performed using the Newman–Keuls post hoc test to identify specific between‐group differences. All statistical analyses were conducted using SPSS 20.0 software and Prism 9.0 software. All the experimental data were presented as the mean ± standard deviation (mean ± SD), with a probability value of *p* < 0.05 considered statistical significance.

## 3 Results

### 3.1 Prediction of Potential Targets and Mechanisms of Action for the Anti‐Aging Effects of *Gastrodia elata* via Network Pharmacology and Molecular Docking

**FIGURE 1:**
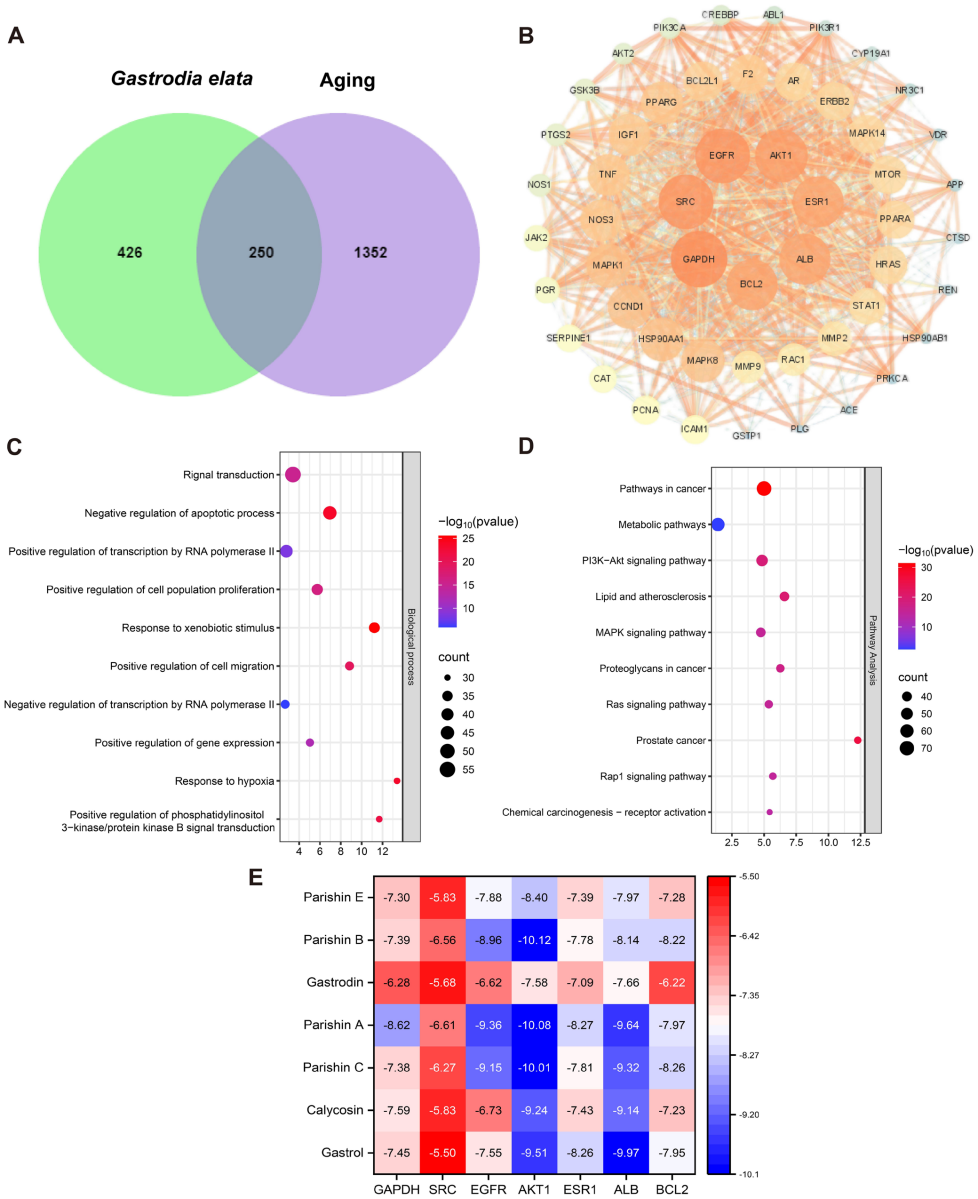
Prediction of potential targets and mechanisms of action for the anti‐aging effects of *Gastrodia elata*. (A) A Venn diagram was used to exhibit the overlapping targets between the active component targets of *Gastrodia elata* and aging‐associated targets. (B) PPI network of anti‐aging intersection targets for active components of *Gastrodia elata*. (C) GO enrichment analysis. (D) KEGG pathway enrichment analysis. (E) Heatmap of molecular docking scores between the key anti‐aging active components of *Gastrodia elata* and key targets.

A total of 156 components of *G. elata* were initially collected through database searches (TCMIP, SymMap, etc.) and literature retrieval. After screening based on absorption, distribution, metabolism, and excretion (ADME) criteria, 27 active components of *G. elata* were identified (Table 1). After sorting and removing duplicates, a total of 676 potential targets of the active components of *G. elata* were obtained. A total of 1602 potential aging‐related targets were obtained from the GeneCards, OMIM, and DrugBank databases. The potential anti‐aging targets of *G. elata* were 250 (Figure 1A). To investigate the interaction between the targets of *G. elata* active components and disease targets, 250 intersection targets were imported into the STRING database. Isolated and marginal targets were removed, and the remaining targets were then imported into Cytoscape 3.9.0 software. The top 7 targets with the highest degree values were selected as key anti‐aging targets of *G. elata* (GAPDH, SRC, EGFR, AKT1, ESR1, ALB, BCL2), and a PPI network was constructed (Figure 1B). The DAVID database was used for GO and KEGG enrichment analyses of the 250 intersection targets to identify the biological pathways significantly affected by the anti‐aging effect of *G. elata* active components. For GO enrichment analysis (Figure 1C), the biological processes (BP) involved in the anti‐aging effect of *G. elata* included negative regulation of apoptotic process, response to xenobiotic stimulus, response to hypoxia, and positive regulation of phosphatidylinositol 3‐kinase/protein kinase B (PI3K/Akt) signal transduction, etc. In terms of KEGG pathway enrichment analysis (Figure 1D), the pathways involved in the anti‐aging effect of *G. elata* included pathways in cancer, metabolic pathways, PI3K‐Akt signaling pathway, lipid and atherosclerosis, MAPK signaling pathway, proteoglycans in cancer, and Ras signaling pathway. To verify the reliability of the identified potential anti‐aging targets of *G. elata* , molecular docking was performed between the top 7 key active components (Gastrol, Calycosin, Parishin C, Parishin A, Gastrodin, Parishin B, Parishin E) and key targets (GAPDH, SRC, EGFR, AKT1, ESR1, ALB, BCL2). The results showed that the docking scores of all key active components with key targets were less than −5 kcal·mol^−1^ (1 cal ≈4.186 J). Parishins exhibited overall lower docking scores with key targets, indicating strong binding activity, with the best binding effects observed with EGFR, AKT1, and ALB (Figure 1E). Subsequently, PyMol 2.3.4 software and Discovery Studio 2016 software were used to visualize the 3D and 2D models of the interactions between Parishin A, Parishin B, Parishin C, Parishin E and EGFR, AKT1, ALB, respectively. The results showed that Parishin A, Parishin B, Parishin C, and Parishin E docked into the binding pockets of the three key target proteins (EGFR, AKT1, and ALB) (Figure S1A–L), EGFR, AKT1, and BCL2 are associated with processes including oxidative stress resistance and apoptosis. This indicated that parishins from *G. elata* can interact with key aging‐related targets, further verifying the accuracy of the above results. Collectively, the results of network pharmacology and molecular docking showed that parishins from *G. elata* are the key anti‐aging active components of *G. elata* , and they may exert anti‐aging effects by suppressing oxidative stress, inflammatory responses, and apoptosis, as well as regulating metabolism.

**TABLE 1:** The active components of *Gastrodia elata*.

No.	Component	CAS number
1	Gastrodin	62,499‐27‐8
2	Parishin A	62,499‐28‐9
3	Parishin B	174,972‐79‐3
4	Parishin C	174,972‐80‐6
5	Parishin E	952,068‐57‐4
6	Parishin J	
7	4‐Hydroxybenzyl alcohol	623‐05‐2
8	3,4‐Dihydroxybenzaldehyde	139‐85‐5
9	Vanillyl alcohol	498‐00‐0
10	Vanillin	121‐33‐5
11	4,4′‐Methylenediphenol	620‐92‐8
12	Vanillic acid	121‐34‐6
13	Adenosine	58‐61‐7
14	*N*_6_‐(4′‐hydroxybenzyl)‐adenosine	110,505‐75‐4
15	4‐Hydroxybenzylamine	696‐60‐6
16	4‐Hydroxybenzyl methyl ether	5355‐17‐9
17	Gastrol	
18	4‐Butoxyphenylmethanol	6214‐45‐5
19	4‐Hydroxybenzyl ethyl ether	57,726‐26‐8
20	4‐Hydroxybenzyl vanillyl ether	
21	2,4‐Bis (4‐hydroxybenzyl) phenol	34,826‐64‐7
22	4‐(4′‐Hydroxybenzyloxy)benzyl methyl ether	
23	Bis (4‐hydroxybenzyl)sulfoxide	189,639‐17‐6
24	Bis‐(4‐hydroxybenzyl) sulfide	38,204‐93‐2
25	β‐sitosterol	
26	Calycosin	20,575‐57‐9
27	4‐Hydroxybenzaldehyde	123‐08‐0

### 3.2 Effect of GEPE on D‐Gal‐Induced Senescence in BV2 Cells

**FIGURE 2:**
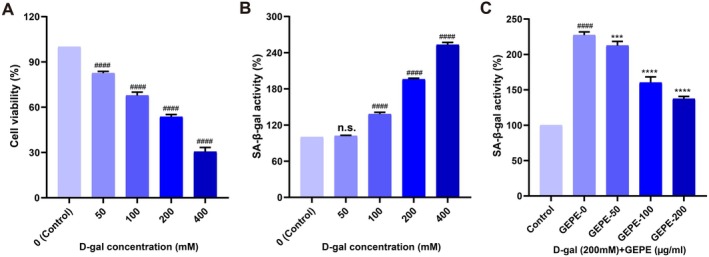
GEPE reduces D‐gal‐induced cellular senescence. (A) BV2 cells were treated with D‐gal at varying concentrations (0, 50, 100, 200, and 400 mM) for a 24‐h incubation period, after which cell viability was assessed using the CCK‐8 assay. (B) BV2 cells were treated with D‐gal at different concentrations (0, 50, 100, 200, and 400 mM) for 24 h and then were determined by SA‐β‐gal assay. (C) BV2 cells were treated with different concentrations of GEPE (0, 50, 100, and 200 mM) and 200 mM D‐gal for 24 h. All experimental data are shown with mean ± SD. n.s. > 0.05, ^####^ *p* < 0.0001 vs. control group, ***p < 0.001, ****p < 0.0001 vs. D‐gal treated cells (GEPE‐0).

Oxidative stress induced by D‐gal can trigger cellular senescence. BV2 cells were treated with varying concentrations of D‐gal, and their viability was assessed. Specifically, cells were exposed to 50, 100, 200, and 400 mM D‐gal for a 24 h incubation period. The findings revealed that cell viability declined remarkably in a concentration‐dependent manner (Figure 2A). Following D‐gal treatment, the activity of SA‐β‐gal—a marker for cellular senescence—in BV2 cells was measured. The results demonstrated that the senescent activity of cells rose as the D‐gal concentration increased. Among the tested concentrations, 200 mM D‐gal was identified as the effective concentration associated with cell viability (Figure 2B) and was thus chosen as the sub‐lethal concentration for all subsequent experimental procedures. Subsequently, the impact of GEPE on cellular senescence was examined using the established senescent cell model. BV2 cells were cotreated with 200 mM D‐gal and GEPE at concentrations of 50, 100, or 200 μg/mL for 24 h. The results indicated that GEPE notably lowered the activity of the SA‐β‐gal senescence marker in a concentration‐dependent way (Figure 2C). These results suggested that GEPE can reduce D‐gal‐induced cellular senescence in BV2 cells.

### 3.3 Effect of GEPE on Behavior and Muscle Strength in Aging Mice

**FIGURE 3:**
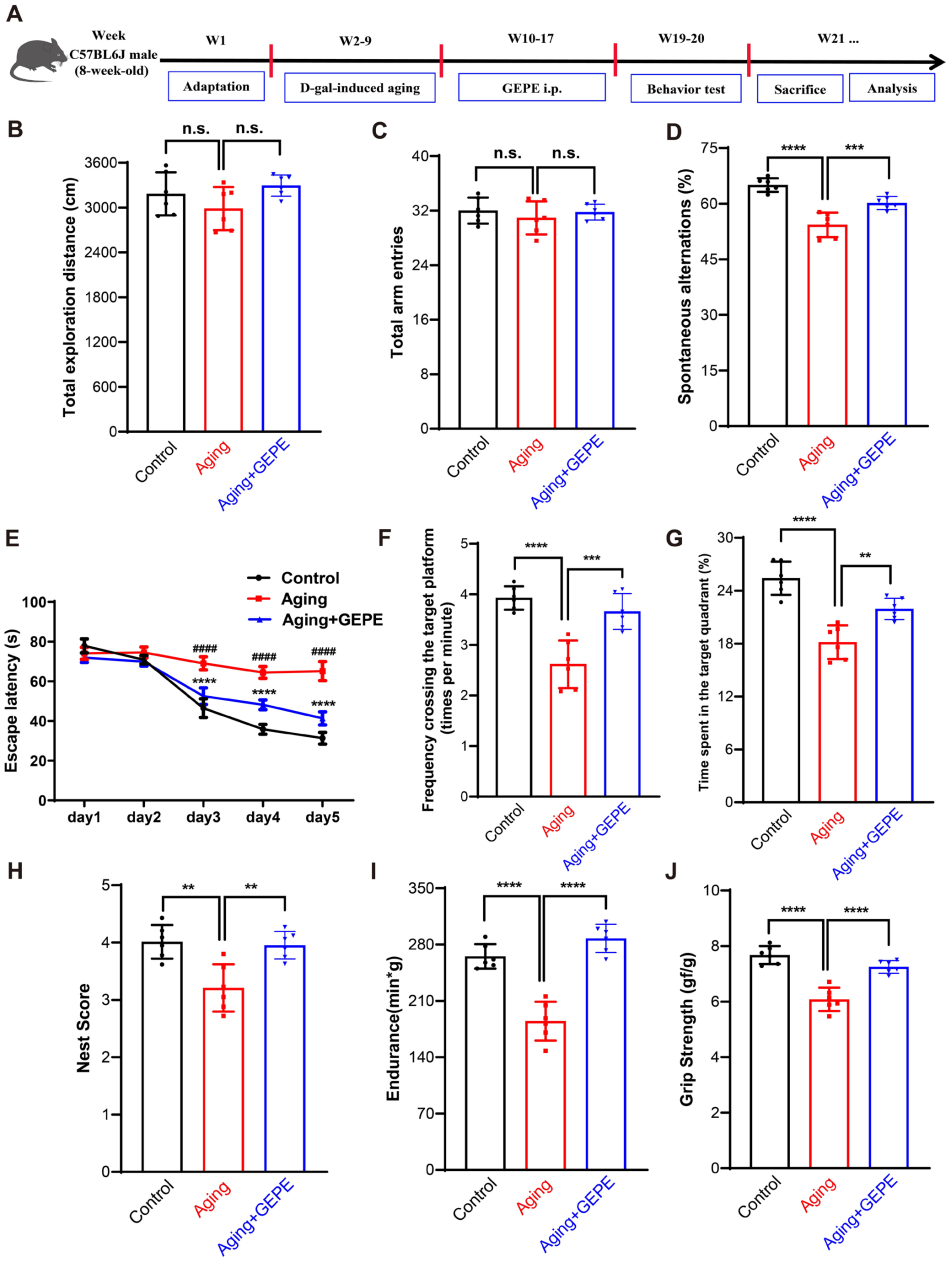
GEPE improves cognitive deficit behaviors and muscle dysfunction in D‐gal‐induced aging mice. (A) Schematic diagram of the experimental design. (B) Total exploration distance in open field test. (C) The number of total arm entries in the Y maze test. (D) The spontaneous alternations in the Y maze test. (E) The latency to the platform of 5 day acquisition training in Morris water maze test. (F) The frequency across the target quadrant in the probe test. (G) The percentage of time spent in the target quadrant in the probe test. (H) The nest score in nest building test. (I) Grip strength. (J) Endurance. All experimental data are shown with mean ± SD. *n* = 6, n.s. > 0.05, ####*p* < 0.0001 vs. control group, **p < 0.01, ***p < 0.001, and ****p < 0.0001 vs. aging group.

To detect whether GEPE exhibits anti‐aging activity in vivo, behavioral experiments were performed according to the experimental design schematic (Figure 3A). In the open field test, no significant difference was observed in the total distance traveled—an indicator of locomotor activity—across the three experimental groups (Figure 3B). For the Y maze test, the total number of arm entries did not differ significantly among the three groups either (Figure 3C). However, when compared with the control group, the spontaneous alternation rate of mice in the aging model group was notably reduced; on the contrary, relative to the aging model group, GEPE treatment led to a significant increase in the spontaneous alternation rate of aging mice (Figure 3D). This indicated that aging impairs the working memory of mice without affecting motor activity, and GEPE improves the age‐related decline in working memory in mice. In the Morris water maze test, during the place navigation training on days 3–5, the latency to locate the hidden platform in the aging model group was significantly longer than that in the control group. Nevertheless, the latency of mice in the GEPE‐treated group was significantly shorter than that in the aging model group (Figure 3E). In the last three place navigation tests, the latency of mice in both the control group and the GEPE‐treated group displayed a downward trend, whereas the latency of mice in the aging model group increased to some degree on day 5. On the 6th day of the experiment, a spatial probe test was performed with the platform taken out. When compared with the control group, the aging model group showed a marked decrease in both the frequency of crossing the area where the platform was previously located and the duration of time spent in the target quadrant. On the contrary, the GEPE‐treated group presented a significant elevation in these two parameters (Figure 3F,G). This indicated that aging impairs the spatial memory of mice, and GEPE plays a positive role in enhancing the spatial memory of aging mice. In the nest building test, when compared with the control group, the nesting score of mice in the aging model group was notably decreased, while GEPE significantly improved these impaired behaviors (Figure 3H). In the grip strength test and endurance test, when compared with the control group, the forelimb grip strength and wire mesh climbing endurance of mice in the aging model group were notably decreased, while GEPE treatment improved this condition (Figure 3I,J). Overall, these behavioral experiments demonstrated that GEPE not only improves D‐gal‐induced cognitive deficit behaviors in aging mice but also ameliorates their muscle dysfunction.

### 3.4 Effect of GEPE on Histopathology in Aging Mice

**FIGURE 4:**
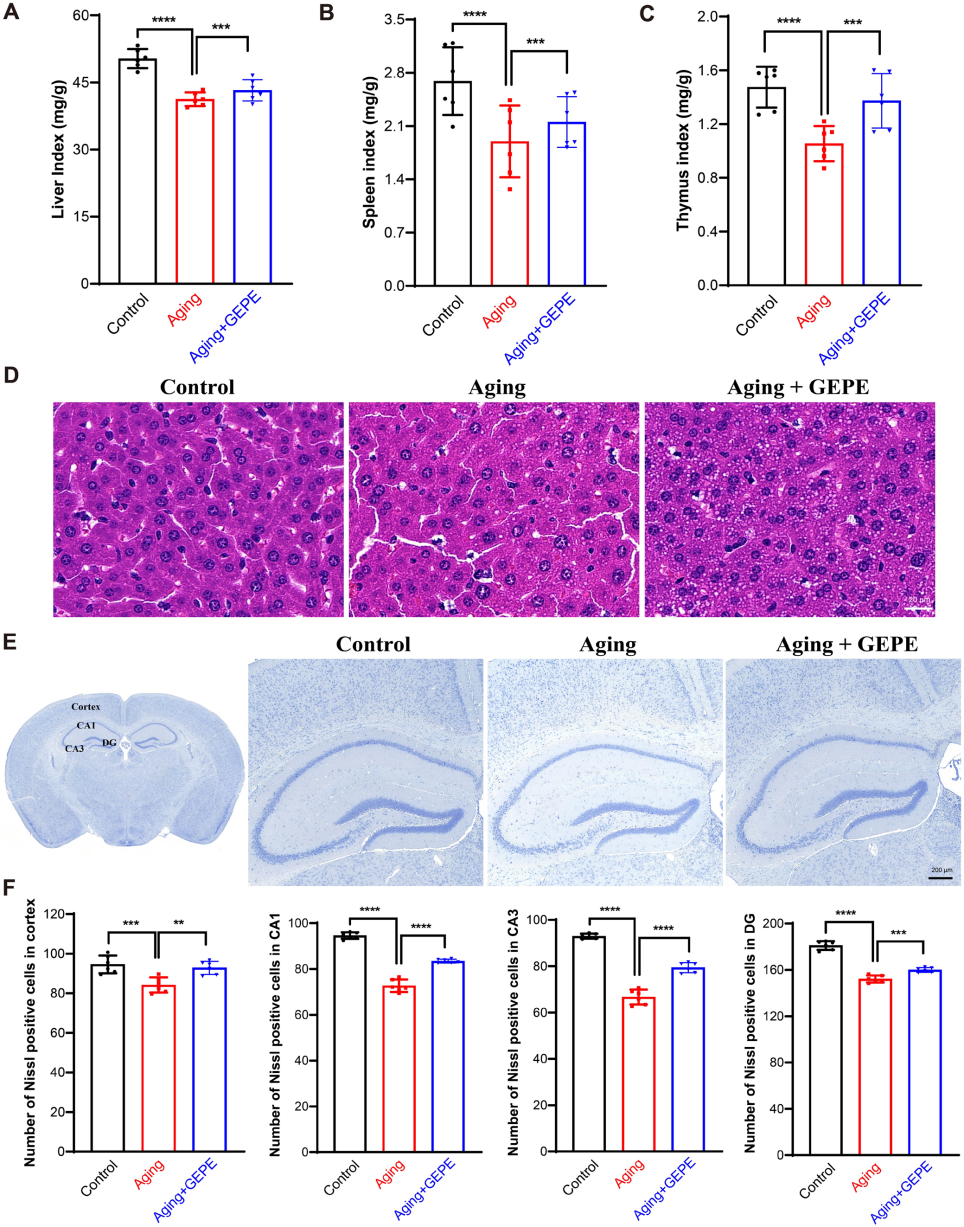
GEPE reverses the pathological changes in D‐gal‐induced aging mice. (A–C) Organ index, liver index (A), spleen index (B), thymus index (C). (D) Representative images of H&E staining of mice liver tissues. (E) Representative images of Nissl staining of mice brain tissues. (F) The quantity of Nissl‐positive cells was determined in the cerebral cortex as well as the CA1, CA3, and DG regions of the hippocampus. All experimental data are shown with mean ± SD. *n* = 6, **p < 0.01, ***p < 0.001, and ****p < 0.0001 vs. aging group.

The organ index reflects the health status of organs. In the present study, the organ indexes of three key organs—the liver, thymus, and spleen—were measured to assess their structural integrity. Results revealed that the liver, thymus, and spleen of mice in the control group maintained optimal morphological conditions, whereas mice in other experimental groups exhibited organ atrophy to different extents. Specifically, when compared with the control group, the organ indexes of the liver, thymus, and spleen in mice belonging to the aging model group were significantly reduced; in contrast, GEPE treatment significantly increased these organ indexes compared with the Aging group (Figure 4A–C). This indicated that GEPE promotes the health of the liver, spleen, and thymus in aging mice, thereby exerting a protective effect against age‐related damage in these animals. To further evaluate hepatic pathological changes, H&E staining was performed on mouse liver tissue samples. The findings revealed that the liver tissue of mice in the control group retained normal structural and morphological features, with no signs of inflammatory cell infiltration. However, in the Aging group, some hepatocytes were loosely distributed, with increased lipid accumulation and inflammatory cell infiltration. GEPE treatment reversed these histopathological changes to a certain extent (Figure 4D). This indicated that GEPE can reduce lipid accumulation and inflammatory responses in the liver of aging mice. Nissl bodies are characteristic structures of neurons, and their number can reflect the growth and development of neurons. To further observe neuronal damage in brain tissue, Nissl staining was performed. The results showed that in the control group, neurons located in the cerebral cortex, hippocampal CA1 region, hippocampal CA3 region, and hippocampal DG region exhibited a dense arrangement and distinct morphological outlines. In the Aging group, the morphology and distribution of cortical and hippocampal neurons were abnormal, with loss of Nissl bodies. GEPE significantly reversed these pathological changes (Figure 4E). Furthermore, quantitative analysis of Nissl‐positive neuron counts revealed that, in comparison to the control group, the number of Nissl‐positive cells in the cerebral cortex and hippocampal region of mice in the aging model group was notably reduced, while GEPE treatment significantly reversed this change (Figure 4F). This indicated that GEPE exerts a neuroprotective effect on aging mice.

### 3.5 Effect of GEPE on Oxidative Stress Status in Aging Mice

**FIGURE 5:**
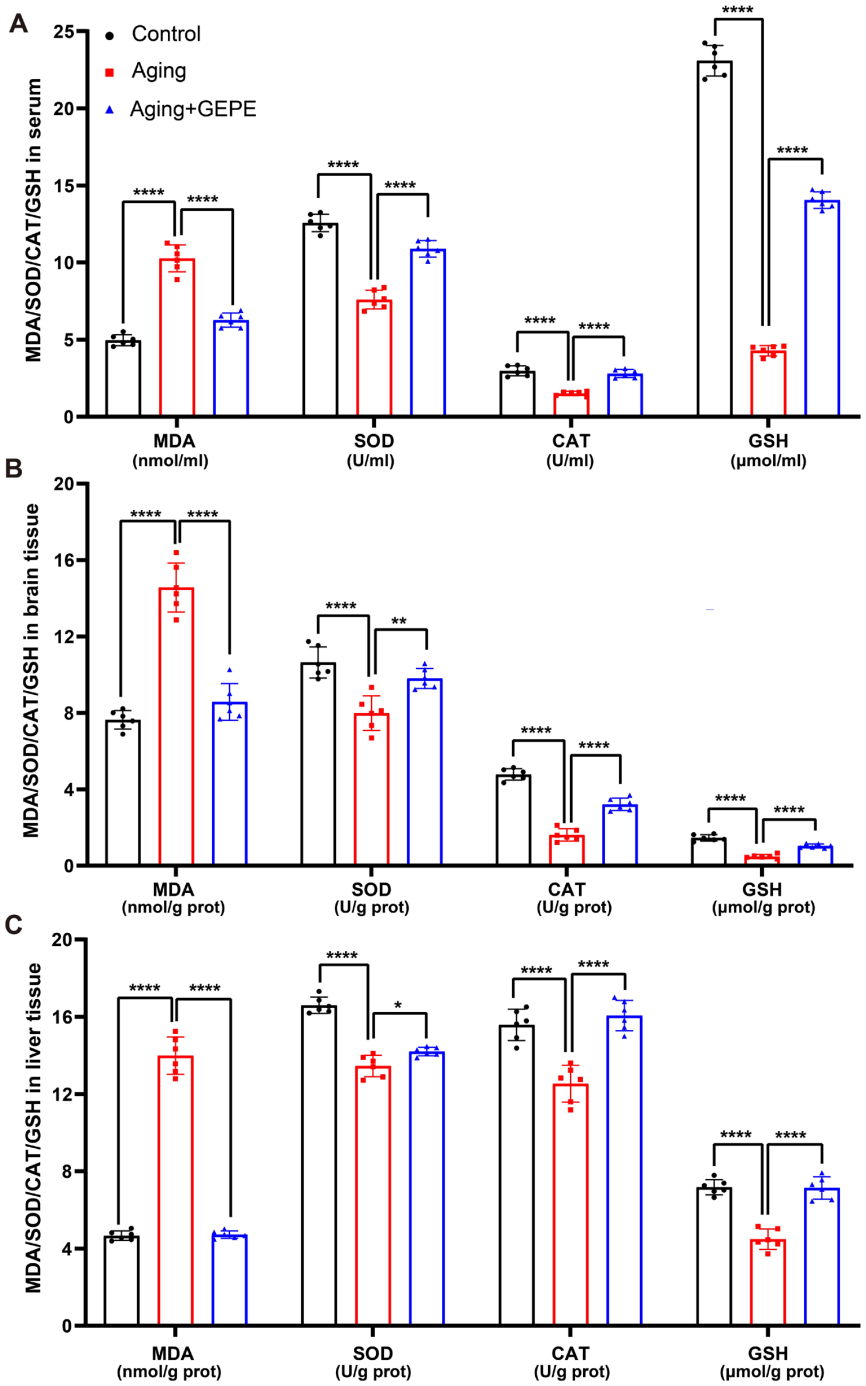
GEPE improves antioxidant stress in D‐gal‐induced aging mice. (A) Levels of MDA, SOD, CAT, and GSH in mice serum. (B) Levels of MDA, SOD, CAT, and GSH in mice brain. (C) Levels of MDA, SOD, CAT, and GSH in mice liver. All experimental data are shown with mean ± SD. *n* = 6, *p < 0.05, **p < 0.01 and ****p < 0.0001 vs. aging group.

Oxidative stress is a typical hallmark of aging. In the present study, the levels of the oxidative marker MDA and the antioxidant indexes SOD, CAT, and GSH in serum, brain, and liver tissues of mice were detected. The results showed that compared with the control group, the MDA—a key marker of lipid peroxidation—in the serum, brain tissue, and liver tissue of mice in the aging model group was notably elevated. In contrast, GEPE exerted a significant inhibitory effect on the rise of MDA levels in aging mice (Figure 5A–C). When compared with the control group, the activities of antioxidant‐related molecules, including CAT, SOD, and GSH, were significantly reduced in the aging model group. However, GEPE intervention led to a marked increase in these antioxidant indicators in aging mice (Figure 5A–C). These results collectively show that GEPE treatment is associated with reduced oxidative stress in serum, brain, and liver tissues of aging mice, with concurrent increases in antioxidant indicators suggesting a potential link.

### 3.6 Effect of GEPE on Inflammation in Aging Mice

**FIGURE 6:**
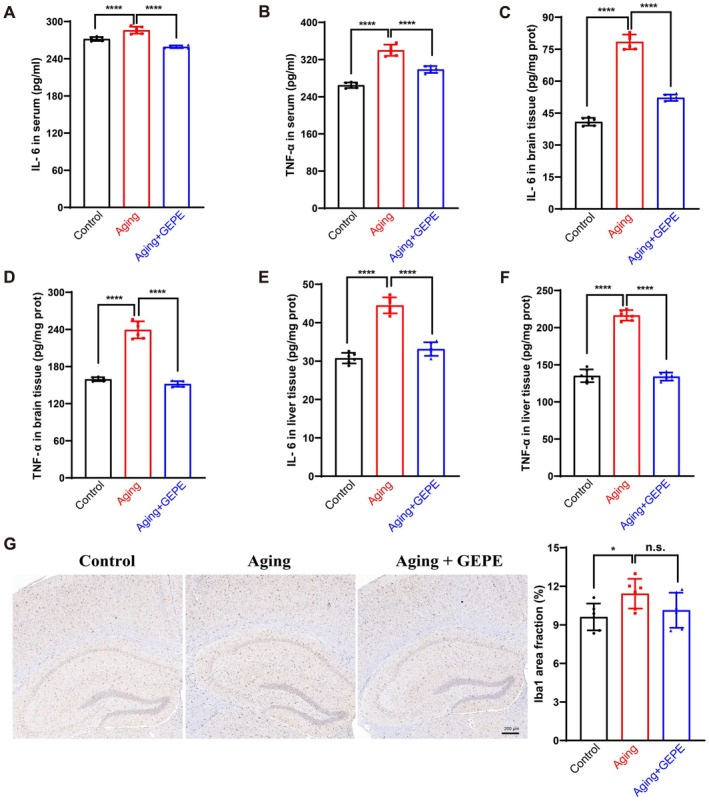
GEPE improves inflammatory factors in D‐gal‐induced aging mice. (A, B) Levels of IL‐6 (A) and TNF‐α (B) in mice serum. (C, D) Levels of IL‐6 (C) and TNF‐α (D) in mice brain. (E, F) Levels of IL‐6 (E) and TNF‐α (F) in mice liver. (G) Immunohistochemical images and quantitative analysis of microglial activation marker Iba‐1 in the hippocampus of mice. All experimental data are shown with mean ± SD. *n* = 6, n.s. > 0.05, *p < 0.05, and ****p < 0.0001 vs. aging group.

The ELISA was employed to determine the expression levels of the inflammatory factors IL‐6 and TNF‐α in the serum, brain tissue, and liver tissue of experimental mice. When compared with the control group, the concentrations of these two inflammatory factors in the serum, brain, and liver tissues of mice in the aging model group were notably elevated; in contrast, GEPE intervention led to a significant reduction in the expression levels of IL‐6 and TNF‐α in aging mice (Figure 6A–F). In addition, microglia are glial cells involved in inflammation and immunity processes within the central nervous system (CNS). In the present study, IHC staining was used to detect the expression of Iba1—a specific biomarker for microglia that responds to oxidative stress and inflammatory stimuli—in the mouse hippocampus. The results demonstrated that relative to the control group, the proportion of the labeled microglial area in the aging model group was significantly higher. However, in comparison to the aging model group, GEPE treatment exerted a certain inhibitory effect on the excessive activation of microglia (Figure 6G). Collectively, these findings suggested that GEPE treatment is linked to alleviated inflammatory responses in aging mice, as evidenced by reduced pro‐inflammatory cytokines and inhibited microglial activation.

### 3.7 Effect of GEPE on Apoptosis in Aging Mice

**FIGURE 7:**
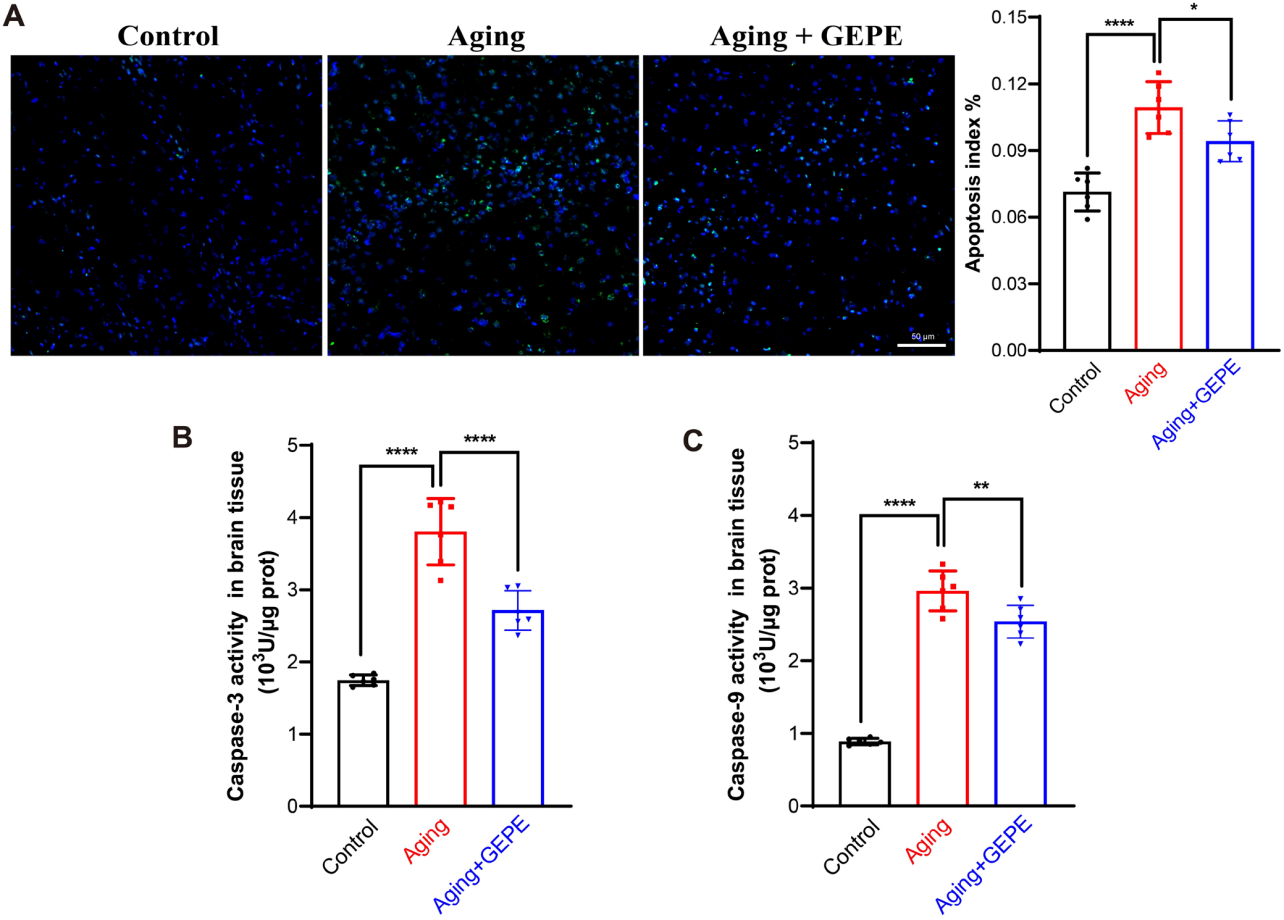
GEPE inhibits excessive apoptosis of brain cells in D‐gal‐induced aging mice. (A) Immunofluorescence images and quantitative analysis of TUNEL staining in mice brain. (B, C) Caspase‐3 (B) and caspase‐9 (C) activity levels in mice brain. All experimental data are shown with mean ± SD. *n* = 6, *p < 0.05, **p < 0.01 and ****p < 0.0001 vs. aging group.

To further explore the regulatory role of GEPE in aging mice, the TUNEL assay was employed to quantify the extent of apoptosis in brain tissue. Results from TUNEL staining demonstrated that, in comparison to the control group, the count of TUNEL‐positive cells in the brain tissue of mice in the aging model group was notably elevated; in contrast, GEPE intervention led to a significant reversal of this apoptotic cell accumulation (Figure 7A). Subsequently, the enzymatic activities of caspase‐3 and caspase‐9—key mediators of the apoptotic pathway—in brain tissue were measured. The findings revealed that relative to the control group, the activities of these two caspases in the brain tissue of the aging model group were significantly heightened. However, GEPE treatment resulted in a marked reduction in the activities of caspase‐3 and caspase‐9 in the brain tissue of aging mice when compared with the aging model group (Figure 7B,C). These results were in alignment with the outcomes of the TUNEL assay, collectively confirming GEPE's inhibitory effect on excessive neuronal apoptosis in aging mice. These results indicated that GEPE treatment can effectively inhibit excessive apoptosis of brain cells in aging mice, thereby delaying the aging process.

### 3.8 Effect of GEPE on Metabolism in Aging Mice

**FIGURE 8:**
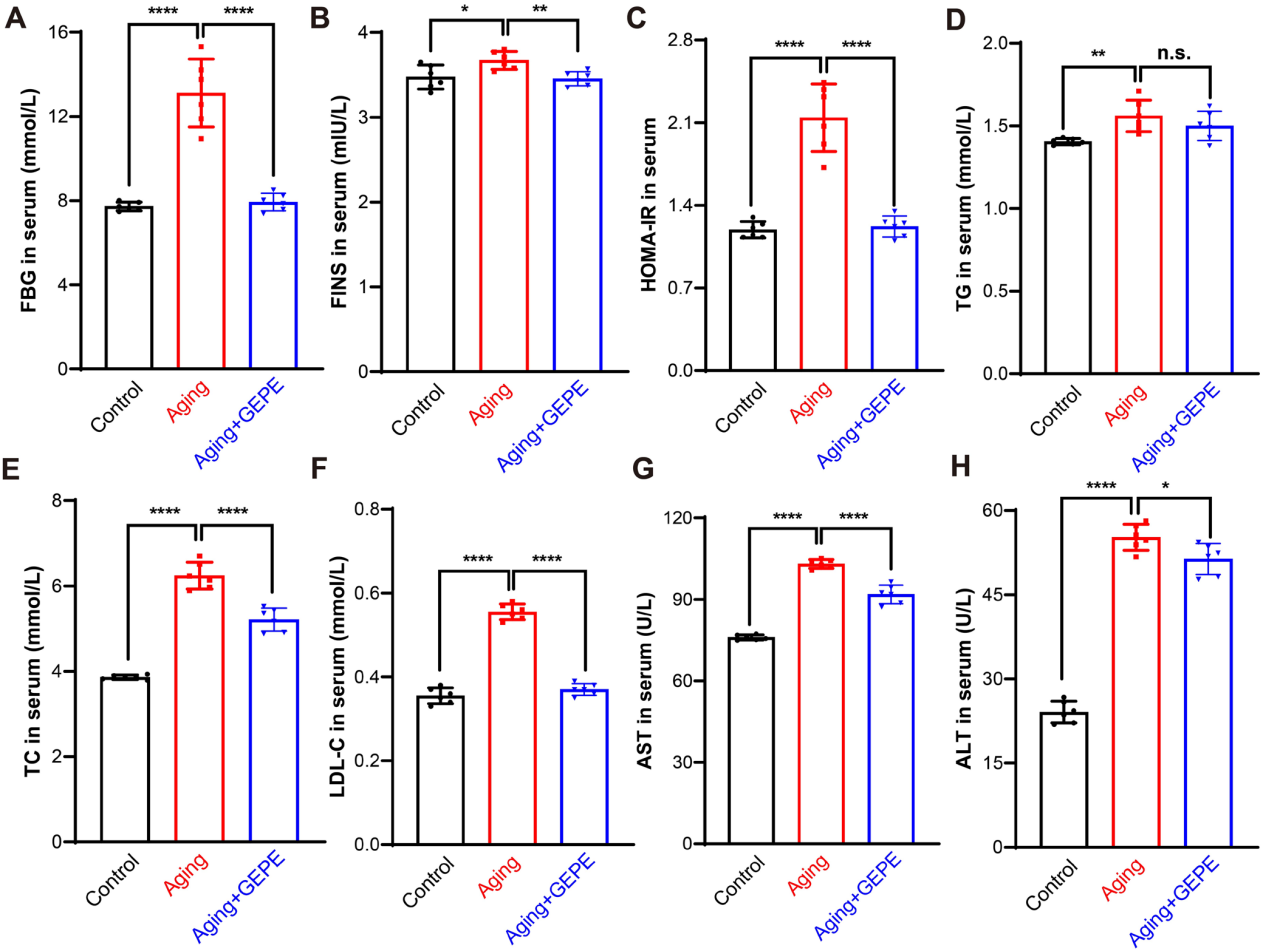
GEPE regulates the metabolic level in D‐gal‐induced aging mice. (A–C) Blood glucose: Levels of FBG (A), FINS (B), and HOMA‐IR (C) in mice serum. (D–F) Lipid metabolism: Levels of TG (D), TC (E), and LDL‐C (F) in mice serum. (G, H) Liver function: Levels of AST (G) and ALT (H) in mice serum. All experimental data are shown with mean ± SD. *n* = 6, n.s. > 0.05, *p < 0.05, **p < 0.01, and ****p < 0.0001 vs. aging group.

Blood biochemical metabolic indicators are commonly used to evaluate the metabolic status and organ function of the body. The impact of GEPE on metabolic processes in aging mice was assessed by measuring alterations in serum indicators related to blood glucose and lipids. For blood glucose assessments, comparisons with the control group revealed that serum levels of FBG, FINS, and HOMA‐IR were markedly higher in the aging model group; in contrast, relative to the aging model group, GEPE administration led to a significant reduction in these three serum indicators in aging mice (Figure 8A–C). This indicated that aging mice are more prone to glucose metabolism disorders, and GEPE intervention contributes to the normalization of blood glucose status in these mice. In terms of lipid metabolism detection, when compared with the control group, the aging model group exhibited substantially elevated serum concentrations of TG, TC, and LDL‐C. However, GEPE treatment resulted in a notable decrease in the serum levels of these three lipid‐related indicators in aging mice when compared with the aging model group (Figure 8D–F). This indicated that aging mice are more prone to lipid metabolism disorders, and GEPE treatment exerts a certain improving effect on the lipid levels of aging mice. Subsequently, the influence of GEPE on hepatic function in aging mice was evaluated by examining variations in serum levels of AST and ALT. The results demonstrated that serum AST and ALT levels were significantly higher in the aging model group than in the control group, while GEPE treatment significantly lowered the serum concentrations of these two liver function markers in aging mice relative to the aging model group (Figure 8G,H). This indicated that aging causes hepatocyte damage, and GEPE treatment can improve liver function in aging mice. These results indicated that GEPE treatment is associated with balanced serum biochemical indicators, normalized lipid metabolism, and improved liver function in aging mice—observations consistent with a potential role in delaying aging.

## 4 Discussion

In this study, using network pharmacology prediction combined with a D‐gal‐induced BV2 microglial cell senescence model and a mouse aging model, we demonstrated for the first time at multiple levels that GEPE treatment is associated with anti‐aging phenotypes, with observations supporting a potential multi‐pathway mechanism involving oxidative stress, inflammatory responses, apoptosis, and metabolic homeostasis. This provides a novel theoretical foundation and experimental evidence to support the development of natural product‐based anti‐aging drugs.

Network pharmacology serves as an efficient analytical tool for investigating the multi‐target regulatory mechanisms of TCM (Zhang et al. 2023; Li, Cui, et al. 2025). In the present study, 27 active components of *G. elata* were screened through multiple databases, and 250 anti‐aging intersection targets were predicted. The key targets such as EGFR, AKT1, and BCL2 are associated with processes including oxidative stress resistance and apoptosis. Additionally, the enriched GO terms (e.g., negative regulation of apoptotic process) and KEGG pathways (e.g., MAPK signaling pathway, metabolic pathways) are closely linked to core aging mechanisms involving inflammation, apoptosis, and metabolism, suggesting that GEPE may exert anti‐aging effects through multi‐pathway synergy. Molecular docking results further confirmed that parishins (Parishin A/B/C) exhibit strong binding activity with key targets such as EGFR and AKT1, and 3D/2D models showed that they can accurately bind to the active pockets of target proteins. This confirms that parishins are the core anti‐aging active components of *G. elata* , provides direction for subsequent experimental verification, and reflects the scientific nature of the “multi‐component, multi‐target” regulation of TCM.

Aging is a systemic and complex temporal change in the organism; therefore, selecting appropriate model organisms and modeling methods is crucial for anti‐aging research. Establishing a cellular senescence model in vitro combined with an aging animal model in vivo is an important approach for preventing or delaying multiple age‐related comorbidities. As mentioned earlier, cellular senescence is a hallmark and major driver of aging (Baker et al. 2016; He and Sharpless 2017; López‐Otín et al. 2023; Xu et al. 2021). During brain aging, senescent microglia gradually accumulate. Studies have shown that oxidative stress induced by D‐gal leads to the accumulation of harmful oxidative products and may cause excessive activation of microglia, resulting in the excessive release of cytotoxic substances and pro‐inflammatory mediators, which accelerates neuroinflammation and neurodegeneration (Gao et al. 2016; Rehman et al. 2017). The vicious cycle of inflammatory responses and oxidative stress may exacerbate neurodegeneration in the brain (Zhao et al. 2017; Choi et al. 2024; Lin and Beal 2006; Kumar et al. 2023; Guzik and Touyz 2017). Therefore, D‐gal‐induced oxidative stress in microglia is a suitable in vitro model for studying aging. In this study, by establishing a D‐gal‐induced BV2 microglial cell senescence model in vitro. In addition, numerous studies have confirmed that long‐term injection of D‐gal can induce aging in organisms, and this model is highly similar to the natural aging of organisms. Therefore, D‐gal is often used to establish animal aging models to explore the potential mechanisms of brain aging and develop corresponding strategies to delay brain aging (Doan et al. 2015; Azman and Zakaria 2019; Su et al. 2025; Kumar et al. 2022; Castelli et al. 2025). Based on this, in vivo, we used D‐gal‐induced aging mice to establish an animal aging model. The core hazard of aging lies in the degeneration of cognitive and physical functions, and behavioral experiments are intuitive indicators for evaluating anti‐aging effects. In this study, aging model mice showed a decreased spontaneous alternation rate in the Y maze test, impaired spatial learning and memory in the Morris water maze test, and reduced nesting ability, muscle strength, and endurance, reflecting the coordinated degeneration of cognitive and physical functions. GEPE intervention significantly improved these behavioral deficits, suggesting that it can improve the healthspan of aging individuals by protecting neural plasticity and maintaining muscle function. Organ indexes and pathological morphology are important indicators for evaluating organ health. GEPE intervention significantly increased organ indices, reduced hepatic lipid accumulation and inflammation, and restored the number of neurons and Nissl body structure in the brain. This multi‐organ protective effect reflects the holistic regulatory advantage of TCM and provides a structural basis for its improvement of cognitive and physical functions.

Aging is a natural phenomenon of living organisms. According to the free radical theory, the accumulation of free radicals beyond the organism's scavenging capacity accelerates cellular senescence, organ dysfunction, and ultimately organismal aging (Golubev et al. 2018). In this study, the D‐gal‐induced aging mouse model exhibited typical characteristics of oxidative stress: an imbalance between oxidation and antioxidant defense. GEPE intervention markedly reversed these oxidative stress‐related indicators. This is consistent with the antioxidant properties of *G. elata* active components, which exert effects by scavenging ROS and activating the antioxidant defense system (Jiang et al. 2014; Zhou et al. 2018; Hsu et al. 2020, 2021; Zhang et al. 2024). This finding suggests a potential association between GEPE treatment, enhanced antioxidant defense capacity, and alleviated oxidative damage, though direct regulatory mechanisms require further validation. Chronic low‐grade inflammation is another important hallmark of aging (Huang et al. 2023; Caldarelli et al. 2024; Morgado et al. 2025). In the present study, mice in the aging model group exhibited a notable elevation in the levels of pro‐inflammatory factors across three types of samples: serum, brain tissue, and liver tissue, and excessive activation of microglia in the hippocampus of mice, indicating coordinated exacerbation of central and peripheral inflammation. GEPE intervention significantly decreased the expression of these inflammatory factors and inhibited excessive activation of microglia, which is consistent with the enrichment results of inflammation‐related pathways (e.g., PI3K‐Akt, MAPK) predicted by network pharmacology. This result indicates that GEPE treatment is linked to modulated central and peripheral inflammation, which may contribute to mitigating the “oxidative stress‐inflammatory activation” vicious cycle, which is consistent with the multi‐site anti‐inflammatory advantage of TCM and provides an inflammatory mechanism explanation for its improvement of cognitive function in brain aging. Moreover, in neural tissue, increased neuronal apoptosis directly leads to cognitive decline (Wang et al. 2023; Qi et al. 2024; Liu et al. 2024). In the present study, a notable elevation was detected in the quantity of TUNEL‐positive cells within the brain tissue of mice in the aging model group, accompanied by increased activities of caspase‐3 and caspase‐9, which serves as an indicator of the activation of the intrinsic apoptotic pathway—also referred to as the mitochondrial pathway. GEPE intervention notably reversed these apoptosis‐related indicators., which was consistent with the neuroprotective results shown by Nissl staining. This regulatory effect may be closely associated with GEPE's inhibition of oxidative stress and inflammation—oxidative damage and inflammatory factors can trigger the apoptotic program by disrupting mitochondrial membrane potential and releasing cytochrome c. The multi‐target effect of GEPE may contribute to mitigating the “oxidative stress‐inflammation‐apoptosis” vicious cycle, a proposed mechanism aligned with the GO functional enrichment result of “negative regulation of apoptotic process” in network pharmacology. Metabolic disorders play a key role in the aging process. In particular, abnormal glucose and lipid metabolism not only accelerates organismal aging but also is closely associated with the onset of age‐related diseases, including neurodegenerative diseases and cardiovascular diseases (Gao et al. 2022, 2025; Wang et al. 2025). In this study, the aging model mice showed obvious glucose metabolism disorders and lipid metabolism disorders, indicating metabolic homeostasis imbalance. GEPE intervention significantly improved the blood glucose and lipid levels of aging mice and decreased the levels of AST and ALT (markers of liver injury), indicating that it can delay aging by regulating glucose and lipid metabolism and protecting liver function. This effect is consistent with the KEGG enrichment results of “metabolic pathways” in network pharmacology, supporting a proposed mechanism where GEPE treatment is associated with restored metabolic homeostasis, potentially linked to enhanced insulin sensitivity, reduced lipid synthesis, and protected hepatocyte function, thereby reducing the accelerating effect of metabolic disorders on aging.

Overall, the present study validates the anti‐aging potential of GEPE and provides experimental evidence for its association with modulated oxidative stress, inflammation, apoptosis, and metabolism—highlighting its value as a natural product‐based candidate. Nevertheless, we acknowledge certain limitations of this work. We only employed a single dose (100 mg/kg) of GEPE for in vivo intervention, and the dose‐dependent effects of GEPE on aging‐related pathways remain unexamined. Additionally, the findings are based solely on the D‐galactose‐induced aging model, with no validation in natural aging models. These limitations will be addressed in future research to further consolidate the anti‐aging potential of GEPE, without affecting the core conclusions of this study.

## 5 Conclusions

This study demonstrates that GEPE treatment is associated with attenuated aging, including enhanced antioxidant capacity, inhibited central‐peripheral inflammation, reduced neuronal apoptosis, and improved metabolic homeostasis—supporting a potential multi‐pathway mechanism for its anti‐aging effects. Future studies can focus on the precise identification of active components, in‐depth analysis of molecular mechanisms, and preclinical translational research, offering preliminary experimental support for the development of natural product‐based multi‐target anti‐aging strategies.

## Author Contributions

**Zhu Li:** conceptualization, methodology, data curation, investigation, formal analysis, funding acquisition, visualization, project administration, writing – original draft, writing – review and editing. **Yue Chen:** conceptualization, methodology, data curation, formal analysis, investigation, writing – original draft. **Huihuang Shi:** software, data curation, investigation, supervision, writing – original draft, visualization. **Peng Tang:** investigation, supervision, writing – original draft, visualization. **Xiangui Zhang:** visualization, writing – original draft.

## Funding

This work was supported by the Yunnan Fundamental Research Projects, 202501AU070176, the open project program of Yunnan Key Laboratory of Gastrodia and Fungi Symbiotic Biology, TMKF2025B08, the Scientific Research Foundation of Yunnan Provincial Department of Education, 2024J1073, and the First‐Class Undergraduate Courses of Zhaotong University, Ztujk202511.

## Conflicts of Interest

The authors declare no conflicts of interest.

## Supporting information


**Figure S1:** fsn371737‐sup‐0001‐FigureS1.docx.

## Data Availability

The data that underpin the findings reported in this study are available from the corresponding author upon the submission of a reasonable request.
