# Supplementary material for: Gastrodia elata–Derived Parishin Extracts Attenuate Aging by Modulating Oxidative Stress, Inflammation, Apoptosis, and Metabolism
Source: Food Sci Nutr. 2026 Apr 3;14(4):e71737. doi: 10.1002/fsn3.71737 (PMC13052268; doi:10.1002/fsn3.71737)
Supplement: Supplementary file 1 — Figure S1: fsn371737‐sup‐0001‐FigureS1.docx. [file FSN3-14-e71737-s001.docx]

**Supplementary Figure**





**Supplementary Figure 1.** The docking modes between the parishins of *Gastrodia elata* and key targets of aging.(A) Binding mode of Parishin A to EGFR. (B) Binding mode of Parishin A to AKT1. (C) Binding mode of Parishin A to ALB. (D) Binding mode of Parishin B to EGFR. (E) Binding mode of Parishin B to AKT1. (F) Binding mode of Parishin B to ALB. (G) Binding mode of Parishin C to EGFR. (H) Binding mode of Parishin C to AKT1. (I) Binding mode of Parishin C to ALB. (J) Binding mode of Parishin E to EGFR. (K) Binding mode of Parishin E to AKT1. (L) Binding mode of Parishin E to ALB.
